# Multicystic Benign Mesothelioma Complicating Pregnancy

**DOI:** 10.1155/2015/687183

**Published:** 2015-08-09

**Authors:** V. A. Tamhankar

**Affiliations:** Department of Obstetrics and Gynaecology, Jessop Wing, Sheffield Teaching Hospitals NHS Foundation Trust, Sheffield S10 2SF, UK

## Abstract

Multicystic benign mesothelioma (MBM) is a rare peritoneal pathology typically affecting women in reproductive age. Though MBM is considered benign, these lesions are prone to recurrence and their growth could be modulated by the presence of oestrogen receptors. Acute presentation of MBM is still very rare in pregnancy and management options are not established. We describe a case of MBM presenting in early pregnancy with acute pain. This was successfully treated with surgical resection. Pregnancy continued uneventfully to term and no evidence of recurrent MBM was found at Caesarean section.

## 1. Introduction

Multicystic benign mesothelioma (MBM) is a rare pathology typically presenting as cystic lesions in the peritoneal cavity [1–4]. These cysts originate from the peritoneal mesothelium. The disease predominantly occurs in women in their reproductive age but rare cases have been described in men [3, 5, 6]. The aetiology of the disease is ill-understood. Some consider it to be a reactive process secondary to previous surgical trauma or inflammation causing peritoneal inclusion cysts [4, 7]. Others have considered a neoplastic aetiology particularly with the associated risk of recurrence after resection [1, 3, 8–11]. Sawh et al. have shown that a proportion of MBM are oestrogen receptor carriers and hence their growth and progress may be dependent on hormonal influence [2]. Such hormone sensitivity of MBM has been targeted as a therapeutic option with some success [12, 13].

MBM remains a poorly understood condition with less than 150 reported cases. Most of the data comes from isolated case reports and short case series. Though this condition predominantly affects women in reproductive age, not a lot is known about its course and management in pregnancy. On review of literature, we found only five reported cases of MBM complicating pregnancy [13–17]. In four of them, MBM was found as an asymptomatic or incidental diagnosis and the fifth case was treated with termination of pregnancy and tamoxifen.

We present a case of MBM who presented with acute pain in early pregnancy and was treated successfully with surgical resection.

## 2. Case Report

**Figure 1:**
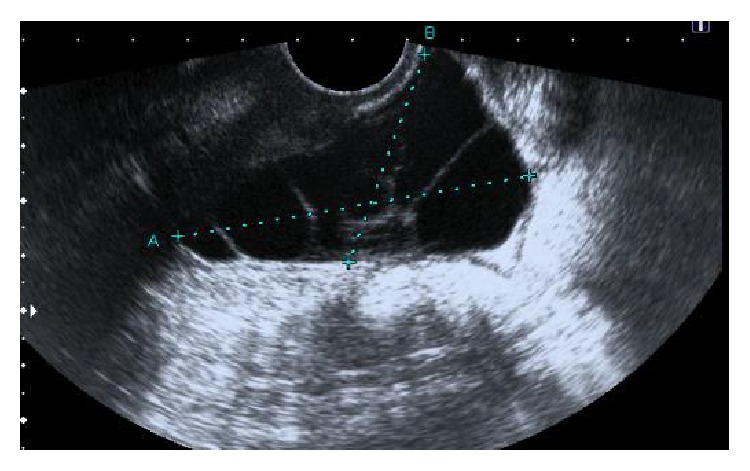
Ultrasound scan image of pelvic multicystic mass.

**Figure 2:**
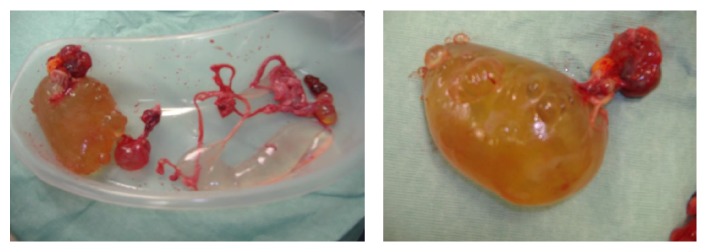
Excised multiloculated cyst with translucent fluid and peritoneal connections.

A 23-year-old nulliparous woman presented at seven weeks of gestation with right iliac fossa pain. She had previous history of appendicectomy but no other known comorbidities except for obesity. Inflammatory blood markers were normal. An ultrasound scan confirmed a viable intrauterine pregnancy, a normal left ovary, and a 6.4 cm × 4 cm multiloculated cystic mass in the pelvis (Figure 1). The right ovary was not visualised separately. The differential diagnoses of a cystic right ovarian mass or MBM were considered. As the initial acute symptoms settled with analgesia, a decision was made to continue expectant treatment in the first trimester. The patient however returned six weeks later with increasing pain. Examination revealed localised peritonitis with tenderness and guarding in the right iliac fossa. A repeat ultrasound scan revealed an increased size of the existing multicystic mass (13 cm × 7 cm). Due to increasing pain, presence of peritoneal signs in the right iliac fossa, and uncertain nature of this cystic mass a decision was made to surgically remove this mass. An exploratory laparotomy was performed at 14 weeks of gestation. The multicystic mass was seen adherent to the pelvic peritoneum and was free from both ovaries, which were normal. There was no evidence of any other inflammatory bowel pathology, pelvic haemoserous fluid, or rupture of the cystic lesion. The lesion was excised (Figure 2) and histology confirmed the diagnosis of MBM. No antibiotics were given postoperatively. The patient recovered from the procedure and continued with the pregnancy uneventfully. She eventually underwent an emergency Caesarean section at term due to failure to progress in labour. At delivery there was no recurrence of the previously noted peritoneal cysts.

## 3. Discussion

MBM is a rare pathology and difficult to diagnose preoperatively. Ultrasound scan, magnetic resonance imaging, and computerised tomography have been used to identify multicystic lesions without adjacent organ invasion or lymphadenopathy. Histological confirmation depends on finding thin walled cysts lined by simple cuboidal or flattened epithelial cells which stain positive for calretinin, a marker of mesothelial origin [2].

MBM is commonly found as an incidental diagnosis or presents with chronic dull pain or mass. Finding MBM cysts in the pelvis along with an intrauterine pregnancy on ultrasound scan does pose a clinical difficulty. It is impossible to predict if the MBM lesions will progress and grow rapidly during pregnancy or will become symptomatic. If the patient presents with acute pain and peritonitis like in our case the decision of surgical resection is easy to make. Though surgical intervention during pregnancy carries some added risk of miscarriage, our patient made a good recovery and completed a full term pregnancy.

The diagnosis of MBM during pregnancy has been reported in five individual patients so far. In three patients, these cysts have been found incidentally at the time of full term Caesarean sections [14, 15, 17]. These patients remained asymptomatic and the pregnancies proceeded uneventfully. van Bijsterveldt et al. describe pregnancy in a patient who had long-standing MBM, previously confirmed on partial resection [16]. This patient had two separate pregnancies, both uneventful and full term, with no change in the MBM on MRI scan. This patient later required further surgical excision, four years after the second delivery, which reconfirmed the diagnosis of MBM. Data from these four patients and their five pregnancies suggest that MBM usually remains asymptomatic and is an incidental diagnosis during pregnancy. Hence an expectant treatment should be offered.

The fifth case report described a patient presenting with acute pain at five weeks of intrauterine gestation and ultrasound diagnosis of pelvic MBM [13]. This was treated with laparotomy and excision. Histology confirmed the diagnosis of MBM with positive oestrogen receptors. The pelvic MBM recurred and was confirmed 6 weeks after initial resection on ultrasound scan. This patient was then offered termination of pregnancy and treated with tamoxifen. MBM remained stable while on tamoxifen treatment.

Study by Sawh et al. has shown that, though uncommon, MBM can be oestrogen receptor positive [2]. In their study only two of the fourteen patients tested positive for oestrogen receptors. We did not study the oestrogen receptors on the resected histology specimen in our patient. Oestrogen receptor positivity will have an impact on the growth and recurrence risk of such MBM in the hyperoestrogenic state of pregnancy as shown by Jerbi et al. [13]. Recurrence rate after MBM resection remains high between 40 and 50% [2, 6, 7, 11, 18]. Clearly in appropriately selected cases tamoxifen does have a therapeutic role [12, 13].

Our above review demonstrates that asymptomatic MBM can be treated expectantly during pregnancy. However it needs to be differentiated from the malignant pelvic cystic tumours. Contrast enhanced and diffusion weighted magnetic resonance imaging has been shown to be useful in predicting the malignant nature of such cystic lesions [19, 20]. Such imaging would be mandatory if a conservative approach is chosen.

Our case is distinct and adds to existing literature as we have demonstrated successful surgical treatment of symptomatic MBM whilst preserving pregnancy. In their review, van Ruth et al. conclude that completeness of resection predicts recurrence risk [21]. We do believe that macroscopic and microscopic completeness of resection reduces the risk of recurrence and hence recommend surgical treatment in appropriately selected cases.
